# Systemic and Urinary Neutrophil Gelatinase-Associated Lipocalins Are Poor Predictors of Acute Kidney Injury in Unselected Critically Ill Patients

**DOI:** 10.1155/2012/712695

**Published:** 2012-10-20

**Authors:** Annick A. Royakkers, Catherine S. Bouman, Pauline M. Stassen, Joke C. Korevaar, Jan M. Binnekade, Willem van de Hoek, Michael A. Kuiper, Peter E. Spronk, Marcus J. Schultz

**Affiliations:** ^1^Department of Intensive Care Medicine, Tergooi Hospitals, Location Blaricum, 1261 AN Blaricum, The Netherlands; ^2^Department of Intensive Care Medicine, Academic Medical Center, University of Amsterdam, 1105 AZ Amsterdam, The Netherlands; ^3^Laboratory for Experimental Intensive Care and Anesthesiology (L**·**E**·**I**·**C**·**A), Academic Medical Center, University of Amsterdam, 1105 AZ Amsterdam, The Netherlands; ^4^Department of Clinical Epidemiology and Biostatistics, Academic Medical Center, University of Amsterdam, 1105 AZ Amsterdam, The Netherlands; ^5^Department of Intensive Care Medicine, Scheper Ziekenhuis Emmen, 7824 AA Emmen, The Netherlands; ^6^Department of Intensive Care Medicine, Medical Center Leeuwarden, 8934 AD Leeuwarden, The Netherlands; ^7^HERMES Critical Care Group, 1105 AZ Amsterdam, The Netherlands; ^8^Department of Intensive Care Medicine, Gelre Hospitals, Location Lucas, 7334 DZ Apeldoorn, The Netherlands

## Abstract

*Background*. Neutrophil gelatinase-associated lipocalin (NGAL) in serum and urine have been suggested as potential early predictive biological markers of acute kidney injury (AKI) in selected critically ill patients. *Methods*. We performed a secondary analysis of a multicenter prospective observational cohort study of unselected critically ill patients. *Results*. The analysis included 140 patients, including 57 patients who did not develop AKI, 31 patients who developed AKI, and 52 patients with AKI on admission to the ICU. Levels of sNGAL and uNGAL on non-AKI days were significantly lower compared to levels of sNGAL on RIFLE_RISK_ days, RIFLE_INJURY_ days, and RIFLE_FAILURE_ days. The AUC of sNGAL for predicting AKI was low: 0.45 (95% confidence interval (CI) 0.27–0.63) and 0.53 (CI 0.38–0.67), 2 days and 1 day before development of AKI, respectively. The AUC of uNGAL for predicting AKI was also low: 0.48 (CI 0.33–0.62) and 0.48 (CI 0.33–0.62), 2 days and 1 day before development of AKI, respectively. AUC of sNGAL and uNGAL for the prediction of renal replacement therapy requirement was 0.47 (CI 0.37–0.58) and 0.26 (CI 0.03–0.50). *Conclusions*. In unselected critically ill patients, sNGAL and uNGAL are poor predictors of AKI or RRT.

## 1. Introduction

Acute kidney injury (AKI) represents a frequent complication in critically ill patients, with high rates of morbidity and mortality [1–5]. AKI requiring renal replacement therapy (RRT) occurs in up to 5% of patients with AKI, in whom the mortality rate approaches 80% [4]. The lack of early biological markers of renal injury prevents timely patient management decisions, including withholding nephrotoxic agents, administration of putative therapeutic agents, and the initiation of RRT.

Recently, neutrophil gelatinase-associated lipocalin (NGAL) has been implicated as an early predictive biological marker of renal injury [6]. NGAL is a ubiquitous 25 kDa protein, covalently bound to gelatinase from human neutrophils, which is normally expressed in very low concentrations in several human tissues, including the kidney [7, 8]. Expression of NGAL increases in the presence of inflammation and injured epithelia, including the kidney after ischemia reperfusion injury and nephrotoxicity [7, 8]. Systemic NGAL appeared to be of diagnostic as well as prognostic value for AKI in previous studies of critically ill patients [9]. However, the role of NGAL in critically ill patients has mainly been studied in highly selected populations, including children and adults after cardiac surgery [10, 11] or after intravenous administration of contrast [12]. In these populations AKI etiology is clear and timing of the insult is often precisely known.

In the present study we evaluated the performance of NGAL in a group of unselected critically ill intensive care unit (ICU) patients, in which AKI etiology and timing are most of the time unclear. Thus, we chose to study a population reflecting daily practice in our centers and tested two hypotheses. First, we hypothesized that NGAL in serum (sNGAL) and urine (uNGAL) can predict AKI 1 to 2 days earlier than the RIFLE criteria in patients who develop AKI after admission to the ICU. Second, we hypothesized that sNGAL and uNGAL predict the need for RRT, in unselected ICU patients.

## 2. Methods

### 2.1. Study Design

This study is a secondary analysis of a multicenter prospective observational cohort study of unselected critically ill patients in 5 multidisciplinary, closed-format ICUs, in which we collected serial serum and urine samples and determined the first day of AKI based on the RIFLE (risk, injury, failure, loss, and end-stage renal disease) classification system [13, 14]. The institutional review board of all participating institutions approved the protocol, and written informed consent was obtained from all patients or next of kin.

### 2.2. Patients

Patients who were older than 18 years, with an expected duration of mechanical ventilation of at least 48 hours and/or an expected length of ICU stay of at least 72 hours, were enrolled within 48 hours of ICU admission. Chronic RRT was an exclusion criterion.

### 2.3. Data Collected

Demographic data, admission diagnosis, reasons to initiate RRT (oliguria or anuria, high sCr/high sUr, or acidosis), acute physiology age and chronic health evaluation (APACHE) II scores and simplified acute physiology scores (SAPS) II [15, 16] were documented in the first 24 hours after admission. Routine laboratory data, including plasma creatinine, were measured daily.

### 2.4. Baseline Renal Function and Definition of AKI

In order to define the baseline renal function we compared the premorbid levels of serum creatinine (sCr) within 1 year prior to ICU admission with the sCr at ICU admission. The lower of these 2 values served as baseline renal function. In case a premorbid sCr was unavailable, baseline renal function was estimated by solving the modification of diet in renal disease (mdrd) equation, with the assumption of a near lower limit of normal glomerular filtration rate (GFR) of 75 mL/min/1.73 m^2^ [17].

The presence of AKI on admission and development of AKI during stay in ICU were scored using the creatinine and urine output criteria of the RIFLE classification system for AKI [13]. In an additional analysis development of AKI during stay in ICU was scored using solely the creatinine criteria. The first day of AKI (the first RIFLE event) was termed AKI day 0; the 2 days prior to this day were termed AKI day 1 and AKI day 2, respectively.

### 2.5. Sampling and Measurement of NGAL

Blood and urine sampling for NGAL measurements was performed on days 0, 1, and alternate days until the start of RRT, ICU discharge, or death, whatever came first. Blood samples were drawn into sterile Vacutainer tubes and centrifuged at 1.500 ×g for 10 minutes at 4°C. The supernatants were stored at –80°C. Urine samples were taken from a 3-hour urine collection period after assessment of the urine volume, centrifuged, and also stored at –80°C.

All samples of NGAL were measured batch wise by means of a commercial ELISA (R&D Systems, Abingdon, UK) according to manufactures recommendation. We tested multiple dilutions on the samples and made them in duplicate. To compensate for differences in urine flow rate, we normalized the urinary excretion of NGAL for moles of urinary creatinine (uNGAL_corr._) [18]. In addition, as proposed before [19], we used the ratio of sNGAL to uNGAL in additional analyses.

### 2.6. Effect Size

To find out what sort of effect size was to be expected in this secondary analysis, we calculated the standard error, 0.061, using the actual number of included patients in our study. The accompanying 95% confidence limits were 0.63 to 0.87 given the estimated minimal clinically relevant area under the curve to be at least 0.70.

### 2.7. Statistical Analysis

The first RIFLE event (risk, injury, or failure, using the combination of creatinine and urine output criteria, or the creatinine criterion alone) served as the primary endpoint. Initiation of RRT served as a secondary endpoint. Data were analyzed using the Statistical Package for the Social Sciences (SPSS) for Windows, version 17.0 (SPSS, Chicago IL, USA).

 Continuous variables were expressed as mean (±SD) or median with (IQR). Categorical variables were expressed as counts and percentages. Normally distributed variables were compared using one-way analysis of variance with Bonferroni's correction for multiple comparisons. For significant findings, post hoc *t*-test was applied. Kruskall-Wallis one-way analysis of variance was used to compare nonnormally distributed variables. Chi-square testing was used to test frequencies between groups. Linear mixed models were used to compare NGAL levels among RIFLE stages. Testing was two-tailed; *P* < 0.05 was considered statistically significant.

Patients with AKI at admission were excluded from the diagnostic analysis and included only in the prognostic analysis. We used day 2 and day 1 in patients who developed AKI with day 0 and day 1 in patients who never developed AKI. This time period was chosen as patients who developed AKI fulfilled RIFLE criteria after 2 (1-2) days.

We assessed sNGAL and uNGAL on their ability to detect AKI or predict need for RRT by calculation of the area under the curve (AUC) of the receiver-operating characteristic (ROC) plot.

## 3. Results

### 3.1. Patients

The original study included 170 patients; 19 patients were excluded because data collection was incomplete. Of the remaining 151 patients, samples were no longer available for analysis of NGAL levels in 11 patients: 3 patients who did not develop AKI, 4 patients who developed AKI, and 4 patients with AKI on admission. Therefore, the final analysis included 140 patients, of whom 57 patients did not develop AKI, 31 patients developed AKI, and 52 patients with AKI on admission, when using the MDRD-based baseline sCr and classifying patients according to the creatinine and urine output criteria of RIFLE. There were no differences in baseline characteristics between the original 170 patients included in the study and 140 patients finally analyzed here.

Baseline demographic data are presented in Table 1. Renal and outcome data are presented in Table 2. Patients who developed AKI were significantly older compared to patients who did not develop AKI. There were no significant differences among the groups in terms of premorbid hypertension, diabetes, or chronic kidney disease. The MDRD based baseline sCr was used in 10% of patients who never developed AKI, 13% of patients who developed AKI, and 29% of patients with AKI on admission.

Patients who developed AKI fulfilled RIFLE criteria after 2 (1-2) days. Continuous venovenous hemofiltration (CVVH) was started in 11 out of 83 patients who presented with or developed AKI (13%). The reason to initiate RRT was oliguria/anuria in 7 patients, acidosis in 1 patient, and high sCr or high sUr in 3 patients. The duration between AKI day 0 and start of RRT was median 1 (0-4) day. In comparison with the non-RRT patient, AKI patients who required RRT had significantly higher APACHE II scores on admission and produced less urine.

### 3.2. Serum and Urine Levels of NGAL

**Figure 1:**
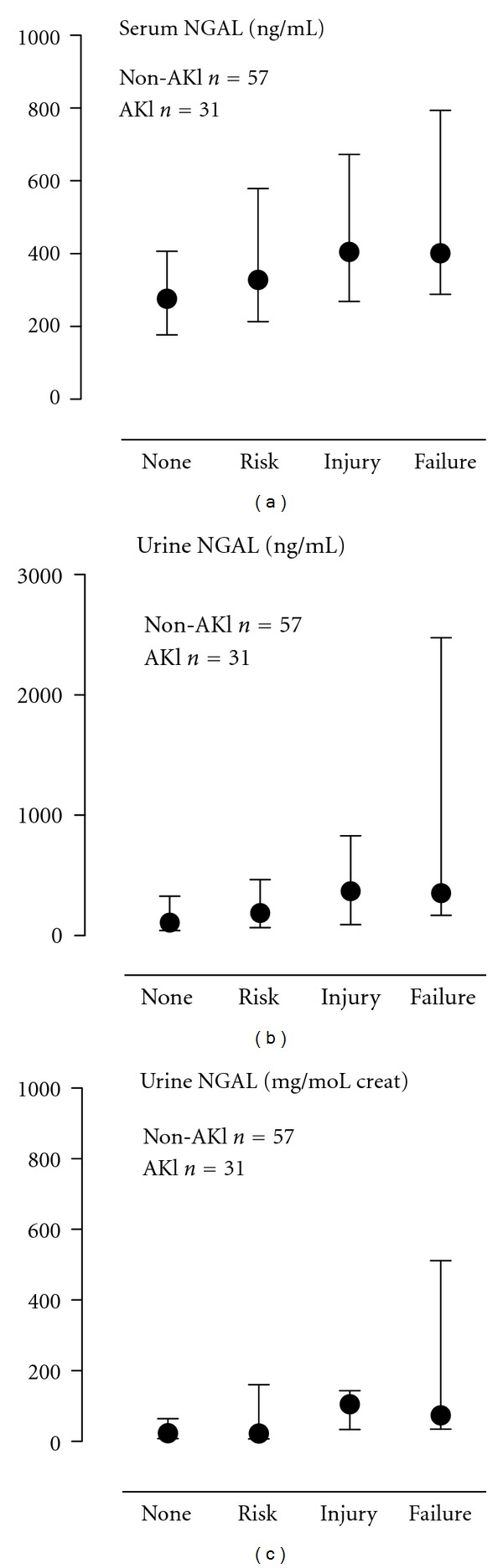
Neutrophil gelatinase-associated lipocalin (NGAL) in serum and urine per RIFLE severity. On nonacute kidney injury (AKI) days NGAL in serum and urine was lower (*P* < 0.05) compared with days fulfilling the RIFLE_RISK_, RIFLE_INJURY_, and RIFLE_FAILURE_ criteria. Non-AKI patients, *N* = 57; AKI patients *N* = 31.

Figure 1 shows levels of sNGAL and uNGAL. Compared to levels of sNGAL on non-AKI days, significantly higher levels of sNGAL were found on RIFLE_RISK_ days, RIFLE_INJURY_ days and RIFLE_FAILURE_ days. Serum NGAL levels were not significantly different among the 3 RIFLE categories.

Similarly, compared to levels of uNGAL on non-AKI days, significantly higher levels of uNGAL were found on RIFLE_RISK_ days, RIFLE_INJURY_ days, and RIFLE_FAILURE_ days. Levels of uNGAL were also not significantly different among the 3 RIFLE categories except for RIFLE_RISK_ versus RIFLE_FAILURE_. Differences in levels of uNGAL per RIFLE category remained similar when urine NGAL was corrected for moles of urinary creatinine.

### 3.3. Prediction of AKI

**Figure 2:**
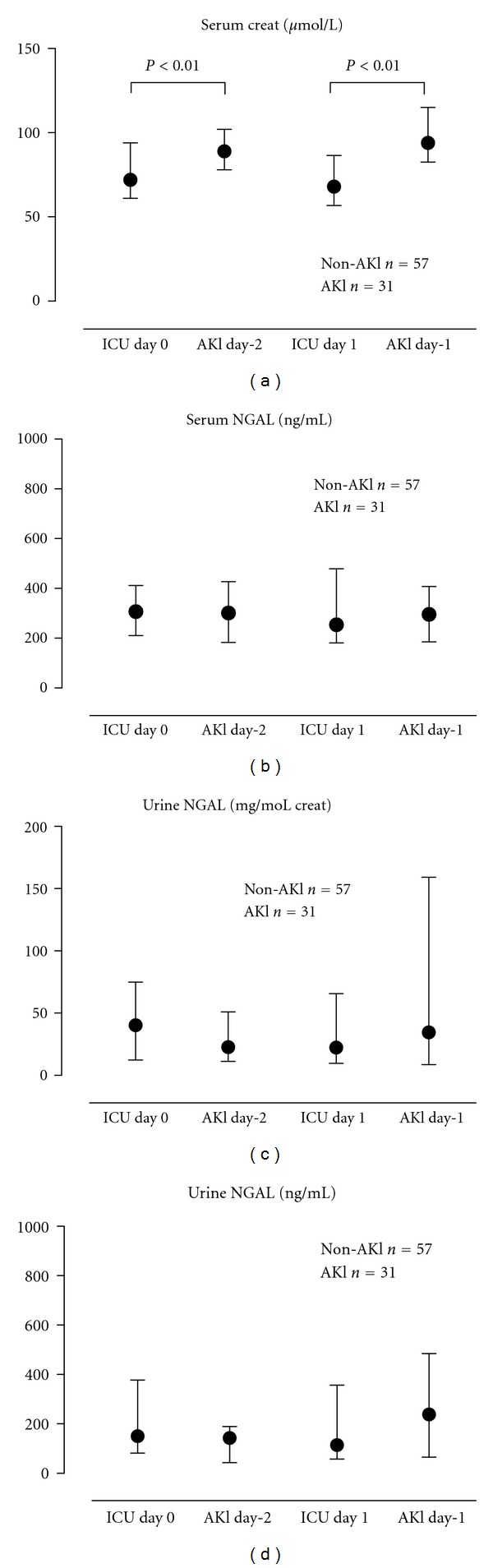
Neutrophil gelatinase-associated lipocalin (NGAL) in serum and urine and creatinine 1 and 2 days prior to acute kidney injury (AKI). Levels are compared to day 0 and day 1 in patients who never developed AKI. Non-AKI patients, *N* = 57; AKI patients, *N* = 31.

We compared levels of sNGAL and uNGAL in the 2 days prior to AKI from patients who developed AKI, with the first 2 study days of admission in patients who did not develop AKI (Figure 2). The areas under the ROC curve of sNGAL and uNGAL for predicting AKI were low and only slightly improved by normalizing the excretion of NGAL for moles of urinary creatinine (Table 3). Similarly, using the ratio of sNGAL to uNGAL the AUC only slightly improved (Table 3).

When we did not use the MDRD-based baseline sCr results were not different. Also, when we only used the creatinine criterion of RIFLE to classify the presence of AKI, and non-AKI and AKI days, areas under the ROC curve of sNGAL and uNGAL for predicting AKI remained low (see electronic supplement).

### 3.4. Prediction of RRT

The areas under the ROC curve of sNGAL and uNGAL for predicting RRT requirement were also low, and did not improve by normalizing the excretion of NGAL for moles of urinary creatinine or when using the ratio of sNGAL to uNGAL (Table 4).

## 4. Discussion

The aims of this prospective multicenter study were to evaluate whether NGAL in serum and urine can detect AKI earlier than the RIFLE criteria in unselected critically ill patients, and whether NGAL in serum and urine can predict RRT requirement. Levels of sNGAL and uNGAL on non-AKI days were significantly lower compared with that on RIFLE_RISK-INJURY-FAILURE_ days. Levels of sNGAL and uNGAL were not different between the patients who finally proceeded with RRT and patients who did not need RRT. The predictive ability of sNGAL and uNGAL was poor, both for detecting AKI and for predicting need for RRT.

Our study is amongst the first studies investigating whether NGAL predicts the development of AKI in unselected critically ill ICU patients in which AKI etiology and timing are often unclear. Our study knows several limitations, though. First, our sample size was relatively small. Second, the MDRD-based baseline sCr had to be used in 18% of our patients. Missing preadmission sCr value is a recognized problem in AKI research, which may lead to misclassification of the incidence of AKI [20]. Contrary, the use of surrogate measures for baseline renal function prevents selection bias. Using MDRD-based baseline, sCr gave us the opportunity to analyze all patients, which otherwise was not possible. Notably, when we did not use the MDRD-based baseline sCr results were not different. Third, the definition of a disease or clinical entity is critical in biomarker research, and the RIFLE criteria may not be an adequate “gold standard” for AKI. The consensus RIFLE definition for AKI is based on sCr and/or urine output. These are functional parameters and may not be appropriate for the detection of injury to the kidney.

We were also the first to use the RIFLE classification with both sCr and urine output, to classify patients. This might have affected our outcomes. Indeed, by using the urine output criterion in addition to the sCr, we may have classified more patients as having AKI. We consider our approach, based upon consensus, more appropriate. Furthermore, when we only used the creatinine criterion of RIFLE, the predictive ability of sNGAL and uNGAL remained poor.

Our results are in contrast with those from other investigations. Indeed, studies investigating sNGAL and/or uNGAL for the prediction of AKI in patients after cardiac surgery [10, 11, 21–27], patients with multiple trauma [28], and patients with sepsis or SIRS [29, 30] showed excellent performances for NGAL as a predictive biological marker of AKI, with areas under the ROC curve of sNGAL and up to 0.91 and of uNGAL up to 0.99. This discrepancy may not come as a surprise, since in these patient groups usually the direct cause of AKI as well as its timing is often obvious. In addition, since collection of specimens in our study could start the next day of admission and specimens were collected on alternate days after the first 2 days, we may have missed peak NGAL levels in our study. However, our approach better reflects daily practice, which is most of the time very different from the ideal research setting.

It should be noted, though, that the results of our study are also different from results from 3 recently published studies that focused on the diagnostic performance of serum NGAL in a more heterogeneous ICU populations [31–33]. Opposite to our findings, these 3 studies suggested sNGAL to be a good and early biological marker of AKI, with increased levels of sNGAL 48 hours before RIFLE criteria were met, and even on admission. Cruz et al. studied 301 consecutive patients admitted to a general medical-surgical ICU [31]. The primary outcome was AKI, defined as an increase in plasma creatinine of at least 50% from baseline or a reduction in urine output to <0.5 mL/kg/hour for >6 hours. sNGAL was a good diagnostic marker for AKI development within the next 48 hours (area under the ROC curve 0.78 (CI 0.65–0.90) and for RRT requirement (area under the ROC curve 0.82 (CI 0.70–0.95). Constantin et al. studied 88 critically ill patients [32]. Focusing on patients without AKI on admission, the area under the ROC curve of sNGAL was 0.96 (CI 0.86–0.99) for prediction of AKI. de Geus et al. studied the predictive value of NGAL in 632 critically ill patients [33]. In this study urine was collected from admission up to 72 hours after admission. The AUC for sNGAL in predicting AKI was 0.77 ± 0.05 (RIFLE_RISK_), 0.80 ± 0.06 (RIFLE_INJURY_), and 0.86 ± 0.06 (RIFLE_FAILURE_); the AUC for uNGAL in predicting AKI was 0.80 ± 0.04 (RIFLE_RISK_), 0.85 ± 0.04 (RIFLE_INJURY_), and 0.88 ± 0.04 (RIFLE_FAILURE_). Several differences in study designs may explain the opposite results. First, Constantin et al. used only one single measurement of uNGAL on admission, since their primary endpoint was the value of NGAL to predict AKI on admission to the ICU [32]. In the studies by Cruz et al. and de Geus et al. patients who already had AKI on admission were not excluded, which may have resulted in higher NGAL levels [31, 33]. Indeed, in the study by Cruz et al. 29% of all patients (67% of all AKI patients) had AKI on admission [31] in the study by de Geus et al. 59% of all patients had AKI on admission [33].

Notably, de Geus et al. found increased uNGAL levels in septic patients without AKI, while levels of sNGAL were not different between patients who developed AKI and those who did not. This is in contrast to a study of adult septic patients by Mårtensson et al. [34]. While in this study the AUC for sNGAL was low (0.67), the AUC for uNGAL was good (0.86). 

As described by Haase et al. [9], the use of a standardized NGAL assay reported a better AUC for NGAL than individually developed research-based assays. Cruz et al. and Constantin et al. used the Triage Meter (Biosite, San Diego) [31, 32]. In our study we used a commercial ELISA. It is uncertain whether this difference explains the differences between our study results and those from other studies.

A large proportion of our patients already had AKI on admission. This is similar to other studies on predictive biomarkers for AKI in a heterogeneous ICU population [31, 33]. It makes little sense to predict AKI in patients who already have AKI, except if NGAL predicts progression in AKI severity, including the need for RRT. Unfortunately, in our study both sNGAL and uNGAL were no predictors of RRT requirement, although it must be mentioned that the number of patients eventually requiring RRT in our study was small. Several factors can be of influence on our results regarding the predictability of need for RRT. For instance, the reason for initiating RRT in our cohort was much more diverse than in patients after cardiopulmonary bypass [10, 11, 22–27].

Nearly 40% of our patients never developed AKI, yet some of these patients had increased levels of NGAL, both in serum and urine. A possible explanation for this finding could be the published reference intervals of sNGAL and uNGAL [35]. They were determined in healthy volunteers with no history of renal disease and may not apply to our critically ill population.

In conclusion, in this multicenter study of unselected critically ill patients, both sNGAL and uNGAL were poor predictive biological markers for AKI. Moreover, sNGAL and uNGAL did not predict the need of RRT.

## Supplementary Material

Supplementary Table 1: Analysis ignoring patients in whom the MDRD based baseline sCr was used.
Supplementary Table 2: Analysis only using the creatinine criterion of RIFLE.

## Figures and Tables

**Table 1:** Group characteristics.

	Patients who never developed AKI	Patients who developed AKI	Patients with AKI on admission	*P* value
	(*N* = 57)	(*N* = 31)	(*N* = 52)	
Age (years)	58.8 (16.1)	67.1 (15.8)	74.4 (9.4)	<0.05
Gender (male) (%)	37 (64.9%)	21 (67.7%)	32 (61.5)	0.84
Weight (kg)	75.7 (14.7)	81.6 (14.9)	81.1 (19.7)	0.17
Height (cm)	176.0 (8.4)	175.2 (8.5)	171.2 (17.1)	0.12
APACHE II score	18.5 (9.4)	19.3 (8.3)	23 (11.5)	0.06
SAPS II	35.9 (12)	42.8 (15)	47.1 (14.8)	<0.05
RIFLE baseline sCr (*μ*mol/L)	69.9 (18.7)	77.9 (15.9)	76.3 (19.8)	0.16
sUrea (mmol/L)	7.9 (5.9–11.2)	11.7 (8.1–17.2)	12.4 (8.1–22.4)	<0.05
sCr (*μ*mol/L)	62 (50–78)	86 (72–104)	110 (73–177)	<0.05
sNGAL (ng/mL)	269 (180–398)	307 (187–460)	343 (238–652)	<0.05
uNGAL (ng/mL)	99 (41–301)	149 (52–405)	289 (92–602)	<0.05
uNGAL_corr_ (mg/molCr)	23 (9–64)	40 (13–142)	23 (8–101)	0.05
Primary diagnosis (*n*) (%)				
CPB	1 (1.7%)	2 (6.4%)	—	0.25
Cardiovascular failure	1 (1.7%)	4 (12.9%)	4 (7.7%)	0.11
Cerebrovascular event	2 (3.5%)	0 (0%)	0 (0%)	—
Hemorrhagic shock	7 (12.3%)	1 (3.2%)	5 (9.6%)	0.37
Multiple trauma	3 (5.3%)	2 (6.4%)	1 (1.9%)	0.55
Elective major surgery	1 (1.8%)	1 (3.2%)	5 (9.6%)	0.15
Respiratory failure	22 (38.6%)	10 (32.2%)	14 (26.9%)	0.43
Septic shock	20 (35.1%)	11 (35.5%)	24 (46.2%)	0.44
Admission category (*n*) (%)				
Medical	27 (47.4%)	14 (45.2%)	21 (40.4%)	0.76
Surgical	30 (52.6%)	17 (54.8%)	31 (59.6%)	0.76
Days from admission until AKI (median and range)	NA	2 (1-2)	0	—
Worst AKI score in ICU (*n*) (%)				
Risk	—	25 (80%)	22 (42%)	<0.05
Injury	—	4 (12%)	13 (25%)	0.19
Failure	—	2 (6%)	17 (32%)	0.006
CVVH	1 (2%)	3 (10%)	8 (15%)	<0.05
LOS (days)	5 (3–8)	8 (5–18)	6 (3–9)	0.001
ICU mortality	0	4 (13%)	8 (15%)	<0.05
Hospital mortality	4 (7.0%)	5 (16.1%)	15 (29%)	<0.05

**Table 2:** Characteristics of acute kidney injury patients by presence or absence of CVVH.

	CVVH	no CVVH	*P* value
	(*N* = 11)	(*N* = 72)	
Age (years)	68.9 (11.6)	72.1 (12.7)	0.43
Gender (male)	7 (63.6%)	46 (63.9%)	0.99
APACHE II score	28 (20–30)	19 (14–25)	0.05
SAPS II	51 (44–57)	45 (35–51)	0.11
sUrea (mmol/L)	11 (7–23)	10 (7–18)	0.24
sCr (*μ*mol/L)	147 (94–299)	100 (76–139)	0.03
sNGAL (ng/mL)	338 (251–798)	341 (205.4–622)	0.56
uNGAL (ng/L)	303 (39–750)	219 (92–535)	0.90
uNGAL_corr._ (mg/mol Cr)	38 (7–342)	34 (9–114)	0.51
Urine output (mL/day)	1480 (493–1960)	2535 (1826–3792)	<0.001
AKI prior to CVVH (days)	1 (0–4)	NA	—

**Table 3:** AUC (CI) for sNGAL, uNGAL, and uNGAL_corr._ in predicting AKI.

	Day 2	Day 1
sNGAL	0.45 (0.27 to 0.63)	0.53 (0.38 to 0.67)
uNGAL	0.48 (0.33 to 0.62)	0.48 (0.33 to 0.62)
uNGAL_corr._	0.47 (0.29 to 0.66)	0.65 (0.51 to 0.79)
sNGAL/uNGAL	0.60 (0.41 to 0.80)	0.47 (0.31 to 0.63)

**Table 4:** AUC (CI) for sNGAL, uNGAL, and uNGAL_corr._ in predicting RRT.

sNGAL	0.47 (0.37 to 0.58)
uNGAL	0.26 (0.03 to 0.50)
uNGAL_corr._	0.27 (0.0 to 0.57)
sNGAL/uNGAL	0.26 (0.01 to 0.51)
