# Supplementary material for: Systemic and Urinary Neutrophil Gelatinase-Associated Lipocalins Are Poor Predictors of Acute Kidney Injury in Unselected Critically Ill Patients
Source: Crit Care Res Pract. 2012 Oct 20;2012:712695. doi: 10.1155/2012/712695 (PMC3483834; doi:10.1155/2012/712695)
Supplement: Supplementary file 1 — Supplementary Table 1: Analysis ignoring patients in whom the MDRD based baseline sCr was used. Supplementary Table 2: Analysis only using the creatinine criterion of RIFLE. [file 712695.f1.pdf]

ARO 100612

ELECTRONIC SUPPLEMENT TO:

Systemic and Urinary Neutrophil Gelatinase–Associated Lipocalin are Poor Predictors of Acute Kidney Injury in Unselected Critically Ill Patients

Annick A. Royakkers[1,2], Catherine S. Bouman[2], Pauline M. Stassen[3], Joke C. Korevaar[4], Jan M. Binnekade[1], Willem van de Hoek[5], Michael A. Kuiper[6,7], Peter E. Spronk[7,8], Marcus J. Schultz[2,3,7]

Tergooi Hospitals, location Blaricum, Blaricum, the Netherlands:
[1]Department of Intensive Care Medicine

Academic Medical Center, University of Amsterdam, Amsterdam, the Netherlands:
[2]Department of Intensive Care Medicine
[3]Laboratory for Experimental Intensive Care and Anesthesiology (L·E·I·C·A)
[4]Department of Clinical Epidemiology and Biostatistics

Scheper Ziekenhuis Emmen, Emmen, the Netherlands:
[5]Department of Intensive Care Medicine

Medical Center Leeuwarden, Leeuwarden, the Netherlands:
[6]Department of Intensive Care Medicine

[7]HERMES Critical Care Group, Amsterdam, The Netherlands

Gelre Hospitals, location Lucas, Apeldoorn, the Netherlands:
[8]Department of Intensive Care Medicine

Correspondence
Annick A.N.M. Royakkers
Department of Intensive Care
Academic Medical Center
Meibergdreef 9
1105 AZ Amsterdam
The Netherlands
Tel: 0031205669111
E–mail: aro@hetnet.nl

Analysis ignoring patients in whom the MDRD based baseline sCr was used

With the use of the MDRD based baseline sCr the "surrogate" baselines might have influenced the results of our analysis. In the reported analysis the MDRD was used in 6 patients in the group who never developed AKI, and in 5 patients who developed AKI.

We repeated the analysis ignoring these patients. Leaving out patients in whom the MDRD based baseline sCr was used did not improve the predictive ability of sNGAL and sNGAL (table 1).

Table 1. AUC (CI) of sNGAL, uNGAL (ignoring patients in whom MDRD based baseline sCr was to be used) in RRT and AKI

	RRT	AKI	
		Day -2	Day -1
sNGAL	0.50 (0.39-0.61)	0.49 (0.26-0.71)	0.54 (0.40-0.69)
uNGAL	0.27 (0.03-0.51)	0.37 (0.19-0.56)	0.51 (0.36-0.66)

Analysis only using the creatinine criterion of RIFLE

The use of the RIFLE classification with both sCr and urine output, to classify patients could be considered to give misclassification of patients in having AKI. Therefore we performed an additional analysis only using the creatinine criterion, ignoring urine output.

With this approach 71 patients did not develop AKI, 33 patients developed AKI during admission and 36 patients had AKI at admission. When we only used the creatinine criterion of RIFLE to classify the presence of AKI, and non–AKI and AKI

days, areas under the ROC curve of sNGAL and uNGAL for predicting AKI remained low (table 2).

Table 2. AUC (CI) of sNGAL, uNGAL (we only used the creatinine criterion of RIFLE) in RRT and AKI

	RRT	AKI	
		Day -2	Day -1
sNGAL	0.42 (0.28 to 0.56)	0.44 (0.24 to 0.64)	0.45 (0.30 to 0.60)
uNGAL	0.35 (0.10 to 0.59)	0.39 (0.21 to 0.58)	0.59 (0.42 to 0.75)
